# Two are Better Than One: Valuing Medical Friendship

**DOI:** 10.5041/RMMJ.10194

**Published:** 2015-04-29

**Authors:** John H. Davidson, Shraga Blazer

**Affiliations:** 1 Assistant Professor of Medicine, Mayo Medical School, and Consultant, Division of Executive and International Medicine, Mayo Clinic, Rochester, MN, USA; 2 Editor-in-Chief, Rambam Maimonides Medical Journal and Director of the Department of Neonatology and Neonatal Intensive Care Unit, Rambam Health Care Campus, Haifa, Israel, and the Ruth & Bruce Rappaport Faculty of Medicine, Technion, Israel Institute of Technology, Haifa, Israel

Dear Friends and Colleagues,

*Tovim hashnaim min ha-echad* *…* Two are *better* than one; because they have a good reward for their labor. For if they fall, the one will lift up his friend: but woe to him who is alone when he falls; for he has not another to help him up.

*Kohelet*/Ecclesiastes 4:9-10

Our evolved ability, our psychological and biological capacity, to make friends even with strangers is a defining characteristic of our species, an evolved human trait marking us apart from most other species on earth just as surely as … walking upright on two legs, having opposable thumbs and a prominent chin, *and* possessing the powers of both speech and complex abstract reasoning … our friendships … connect us with one another in ways that create astonishingly far-reaching social networks capable of transmitting vitally useful information, mobilizing people to action, and in other ways, too, buffering us, our families, and our communities against the trials and tribulations of life.^1^

The post-exilic biblical voice of *Kohelet* above is echoed, even if unintentionally, by that of contemporary anthropologist, ethnologist, and social network analyst, John Edward Terrell, a Chicago Field Museum of Natural History curator.^1^ Both are exemplified by articles in this issue of *Rambam Maimonides Medical Journal* that were inspired by and composed in the wake of a visit almost a year ago (May 2014) by clinicians and scholars from the Mayo Clinic and the University of Minnesota with Israeli colleagues on the Rambam Health Care Campus in Haifa.

It was a day of formal conferences, informal conversations, a facility tour, and shared meals. It was also a day of friend-making, of creating “far-reaching social networks,” as “useful information” was exchanged and “complex abstract” ideas were debated.^1^ A mutuality of support was present among the American and Israeli caregivers and scientists who shared an immersion in the daily challenges of the professional practice of medical science in our different settings. Valuing medical friendship in this tangible way of dialogical teaching, debating, and personal presence, birthed the creative products of mind reflected in the articles of this issue written by the Mayo and Rambam experts.

The Mayo articles include a survey by Lerman et al. of the promise, challenges, and imperative of international collaboration among medical clinicians and investigators.^2^ Bostwick offers an insightful analysis and unique approach to the assessment of suicidality in the general hospital setting.^3^ Fleming posits the relevance of approaching and treating fibromyalgia and other occurrences of medical symptoms of undetermined significance under a rubric of central sensitization syndrome.^4^ Mueller synthesizes the burgeoning literature related to defining, teaching, and assessing professionalism among physicians.^5^ Kreitzer describes the breadth and depth of integrative nursing principles and practice as she has conceived and demonstrated them in the founding of the Center for Spirituality and Healing at the University of Minnesota.^6^ Piderman et al. report the results of a pilot study suggesting the relevance of spiritual life review in the clinical milieu of neurodegenerative illness and brain cancer patients.^7^ Davidson argues for the relevance of literary fiction, and specifically Talmudic legends, in fostering clinical empathy and narrative competence among all caregivers.^8^

The Rambam articles likewise cover a broad range of relevant clinical issues. Klein and his group examine the aftereffects of combat trauma in an important cultural group serving in the Israel Defense Forces—the Bedouins.^9^ The team of Azzam enter the fascinating world of microbiota in the human body, a new and important field of study.^10^ Continuing the pioneering studies generated at Rambam in the field of intrauterine embryonic and fetal ultrasonography, Weiner and his team lay the groundwork for more accurate fetal measurements using three-dimensional sonography.^11^ Admi and colleagues provide an in-depth review on the effects of hospitalization on older adults.^12^ The contribution of Admi and her colleagues and the papers of Kreitzer^6^ and Piderman^7^ from the Mayo group are all particularly noteworthy: They represent the first papers published in *Rambam Maimonides Medical Journal* representing the field of nursing—an integral aspect of clinical practice.

As a whole, these considered pieces are the creative products from that day in May 2014 of valuing medical friendship with the making of “astonishingly far-reaching social networks” among Israeli and American colleagues. The fruitful ethic, medical and otherwise, most embodied by that event is finally captured in the well-known rabbinic saying:

*Aseh l’cha rav, ukneh l’cha haver* *…*

Get yourself a teacher, acquire for yourself a friend with whom to study, and give every person the benefit of the doubt.

*Pirkei Avot/*Ethics of the Fathers 1:6
